# High-resolution extracted ion chromatography, a new tool for metabolomics and lipidomics using a second-generation orbitrap mass spectrometer

**DOI:** 10.1002/rcm.4015

**Published:** 2009-05-30

**Authors:** Albert Koulman, Gary Woffendin, Vinod K Narayana, Helen Welchman, Catharina Crone, Dietrich A Volmer

**Affiliations:** 1 Medical Research Council, Elsie Widdowson LaboratoryCambridge, UK; 2 Thermo Fisher ScientificHemel Hempstead, UK; 3 Thermo Fisher ScientificBremen, Germany

## Abstract

Most analytical methods in metabolomics are based on one of two strategies. The first strategy is aimed at specifically analysing a limited number of known metabolites or compound classes. Alternatively, an unbiased approach can be used for profiling as many features as possible in a given metabolome without prior knowledge of the identity of these features. Using high-resolution mass spectrometry with instruments capable of measuring *m/z* ratios with sufficiently low mass measurement uncertainties and simultaneous high scan speeds, it is possible to combine these two strategies, allowing unbiased profiling of biological samples and targeted analysis of specific compounds at the same time without compromises. Such high mass accuracy and mass resolving power reduces the number of candidate metabolites occupying the same retention time and *m/z* ratio space to a minimum. In this study, we demonstrate how targeted analysis of phospholipids as well as unbiased profiling is achievable using a benchtop orbitrap instrument after high-speed reversed-phase chromatography. The ability to apply both strategies in one experiment is an important step forward in comprehensive analysis of the metabolome. Copyright © 2009 John Wiley & Sons, Ltd.

Metabolomics and lipidomics are relatively new scientific disciplines, currently driven by the performance of the analytical instrumentation used. Advancements in these disciplines are often the result of new developments in mass spectrometry,^1^,^2^ or new tools to interrogate the experimental data.^3^ Metabolomics and lipidomics research usually follows one of two possible strategies.^4^ If metabolic profiling or fingerprinting is essential, data acquisition techniques focus on capturing as many metabolites as possible without initially requiring specific knowledge of the identity of these analytes.^5^ Multi-variate statistics is used to determine the analytes of interest and these are subsequently classified or identified.^6^ This strategy is particularly useful in the search for new compounds, biomarkers, or mechanisms. Some scientists argue that this method is of limited use when data will be integrated with other omics platforms such as proteomics or transcriptomics,^7^ where unidentified analytes obstruct the formation and integration of biochemical networks. For these researchers, it is useful to only collect data on known compounds as they can be interpreted in a biochemical sense.^8^ As the specific, targeted analysis of known compounds demands a different method (e.g., tandem mass spectrometry, MS/MS) than global metabolic profiling (usually full scan MS spectra), the merger of the two strategies would usually result in compromised quality for one or the other of the two strategies. The ability to obtain mass spectra with a very high degree of mass accuracy at sufficient mass resolutions and scan rates opens the possibility for combining both strategies without any such compromises. Analytical instruments capable of high-resolution mass measurements are time-of-flight (TOF), Fourier transform ion cyclotron resonance (FT-ICR) and orbitrap mass spectrometers.

The acquisition of experimental data of sufficient mass resolving power and accuracy has until a few years ago only been possible using FT-ICR-MS, with severe limitations with respect to scan speeds, however. Consequently, FT-ICR instruments have not been routinely applied in high-throughput metabolomics applications. Higher scan speeds are achievable with a TOF analyser but these do not reach the same mass accuracy and resolution.^9^–^11^ For very complex samples, both high resolving power and mass accuracy are required, as available from FT-ICR or orbitrap instruments.^12^–^16^ High-resolution orbitrap mass spectrometry is particular interesting for hyphenated LC/MS applications using ultra-performance liquid chromatography (UPLC) instruments, where sub-2 µm particle columns generate chromatography peaks with peak widths of only a few seconds, requiring at least 2 scans per second to obtain a sufficient number of data points across the peak for quantitation.

Importantly, if high mass accuracy can be maintained in metabolomics applications throughout the duration of the chromatographic experiments, it will be possible to extract ion chromatograms with a sufficiently high degree of accuracy, so that overlapping isobaric signals from salt adducts and lipids containing longer unsaturated fatty acids can be readily separated. Such applications typically require 5 ppm or less mass measurement accuracy.^5^

The aim of the present study is to evaluate a second-generation, benchtop orbitrap mass spectrometry system for application to high-throughput metabolic profiling. Specifically, we are describing the analysis of human plasma samples, with the goal of achieving simultaneous unbiased fingerprinting as well the targeted analysis of large numbers of metabolites within a single run, without compromising the analytical quality of the two strategies used.

## EXPERIMENTAL

### Samples

Plasma samples were collected from 10 healthy volunteers. For each sample, 200 µL aliquots were taken and diluted with either (a) 100 µL of Ringer solution; (b) 40 µL of Ringer solution and 10 µL of palmitic acid stock solution; (c) 40 µL of Ringer solution and 10 µL of glucose stock; (d) 40 µL of Ringer solution and 10 µL of *N*-octanoylsphingosine stock; (e) 40 µL of Ringer solution, 10 µL of palmitic acid stock, 10 µL of glucose, 10 µL of *N*-octanoylsphingosine stock. Also, 250 µL of whole plasma was used, yielding a set of total 60 samples. A volume of 250 µL of each sample was added to 1000 µL of cold acetronitrile. This mixture was centrifuged for 10 min at 13 000 rpm and the supernatant was diluted (1:1) with formic acid (0.1%) and transferred to a 96-well plate ready for analysis by high-performance liquid chromatography (HPLC).

### Chemicals and stock solutions

All chemicals were purchased from Sigma Aldrich (Gillingham, UK) except for *N*-octanoylsphingosine, which was supplied by Cayman Chemical Company (Ann Arbor, USA). Ringer solution was prepared by adding 6.5 g of NaCl, 0.14 g of KCl, 0.065 g of NaH_2_PO_4_, 2 g of glucose, 0.4 g of NaHCO_3_ and 1 mL of 1 M CaCl_2_ to 1 L of H_2_O. Palmitic acid stock solution was prepared by dissolving 6.1 mg of palmitic acid in 0.5 mL of acetonitrile, *N*-octanoylsphingosine stock by dissolving 2 mg of *N*-octanoylsphingosine in 0.5 mL of acetonitrile and glucose stock by dissolving 300 mg of glucose in 0.5 mL of H_2_O.

### Chromatography

Chromatographic separations were performed on a 5.0 × 2.1 mm Hypersil Gold 1.9 µm C_18_ column (Thermo Scientific, Runcorn, UK) using an Accela U-HPLC system (Thermo Scientific, Hemel Hempstead, UK). The column was maintained at 45°C. A binary mobile phase system was used where A = formic acid (0.1%) and B = acetonitrile/isopropyl alcohol (1:1) containing formic acid (0.1%). The mobile phase program at an initial hold (0.0–0.5 min) at 5% B followed by a linear gradient 5–50% B (0.5–5.0 min), then 50–95% B (5.0–5.5 min); the conditions were then held at 95% B (5.5–6.5 min) and returned to the initial conditions (6.5–10.0 min). The total analysis duration was 10 min at a flow rate of 0.25 mL/min. The column eluent was directed to the mass spectrometer.

The 60 samples were run in randomised order and were injected using an injection volume of 10 µL. This sequence was repeated with the same injection volume and then two more times with an injection volume of 5 µL giving a total of 240 consecutive injections.

### Mass spectrometry

Mass spectrometry was performed on an Exactive orbitrap mass spectrometer (Thermo Scientific, Hemel Hempstead, UK) operating in positive ion mode. The heated electrospray (HESI-II) source was used. The sheath gas was set to 20 (arbitrary units) at a temperature of 200°C, the aux gas set to 10 (arbitrary units) and the capillary temperature set to 250°C. The capillary voltage and spray voltage were set to 51 V and 4.2 kV, respectively. The instrument was operated in full scan mode from *m/z* 150–1000 at 50 000 resolving power. The data acquisition rate was 2 Hz. The mass spectrometer was mass calibrated just prior to starting the sequence of 240 injections. All data was acquired using lock mass calibration (*m/z* 214.0896).

### Data analysis

#### Specific analysis of phosphocholine lipids

For the targeted analysis of 50 specific phospholipids (see Table 2), the theoretical exact masses were used with 4 significant figures with a scan width of ±2.5 ppm. The resulting extracted ion chromatograms were integrated and the area-under-the-curve (AUC) was used for relative quantitation. The values were imported into the Dante software,^17^ where missing data was imputed using the k Nearest Neighbour method. Data was further analysed using analysis of variance (ANOVA), principle component analysis (PCA) and partial least-squares data analysis (PLS-DA).

#### MZmine

The raw files were converted into NETcdf files using the Thermo software package Xcalibur. The converted files were imported into MZmine^18^ and peaks were detected using the following settings: noise level = 30000.0; mass resolution = 30000; peak model function = ‘Savitzky-Golay’; min time span = 7.0; *m/z* tolerance = 0.0020. The resulting peak lists were aligned using *m/z* tolerance = 0.0025; retention time tolerance = 10.0%. The resulting peak list was exported as a CSV file and imported into DANTE^17^ where missing data was imputed using the k Nearest Neighbour method, and data was further analysed using ANOVA, PCA and PLS-DA.

## RESULTS AND DISCUSSION

This study evaluates a novel, second-generation orbitrap mass spectrometer for simultaneous targeted and non-targeted lipidomics analysis. The previous generation orbitrap instrument was an expensive, hybrid design, with linear ion trap for MS^n^ front-end prior to high-resolution orbitrap mass analysis. The instrument used in this study is the second-generation, non-hybrid design, consisting of only the orbitrap mass analyser, with mass revolving capabilities of 100 000 at scan repetition rates of 1 Hz. Scanning is possible at higher rates, however, with greatly reduced mass resolving powers. To our knowledge, this is the first report on the analytical performance on this second-generation orbitrap mass analyser.

The data presented in this study was obtained from a single batch of plasma samples consisting of a total of 240 LC/MS runs. The analytical quality of the data was evaluated using several protocols for five selected compounds; the retention times and mass accuracies were checked manually for every 40^th^ sample. The robustness of the mass accuracies was tested by determining the range of the measured *m/z* ratios across the top of the peaks for a width of five scans (for the scan with the highest intensity and two scans each before and after). Table 1 summarizes the mean, standard deviation, and lowest and highest measured *m/z* ratios for the five test analytes. Overall, the observed mass measurement uncertainties using the new orbitrap were comparable to the previous generation orbitrap as well as standard FT-ICR instuments.^19^

**Table 1:** The stability of the mass accuracies across a 240 sample batch. The spread of the measured *m/z* ratios for five selected ions is shown for every 40^th^ run

	Theoretical *m/z*	Average *m/z*	Stdev *m/z*	Min *m/z* observed	Max *m/z* observed	*m/z* spread [ppm]
C_6_H_12_O_6_Na^+^	203.05261	203.05264	0.00008	203.05255	203.05281	1.3
C_16_H_35_O_2_NH^+^	274.27406	274.27399	0.00003	274.27396	274.27405	1.2
C_24_H_38_O_4_H^+^	391.28429	391.28433	0.00012	391.28415	391.28455	1.0
C_24_H_51_O_7_NP^+^	496.33977	496.33991	0.00027	496.33945	496.340564	2.3
C_46_H_81_O_8_NP^+^	806.59643	806.56943	0.00044	806.56830	806.57050	2.7

**Figure 1:**
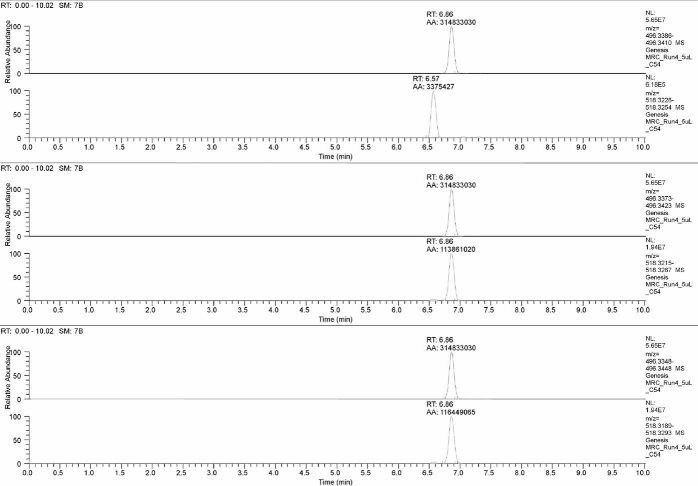
Extracted ion chromatograms for *m/z* 496.3398 and 518.3241. The top two traces are shown for ±2.5 pm windows, middle two traces for ±5.0 ppm and bottom traces for ±10 ppm. Only using the ±2.5 ppm windows, the extracted ion chromatogram for *m/z* 518.3241 is specific for GPCho (18:3/0:0), while the larger window shows an additional signal for the Na^+^ adduct of GPCho (16:0/0:0).

The stability of the measurements across the 240 runs demonstrated that the observed mass accuracies were sufficiently stable to extract ion chromatograms based on very narrow mass windows. The results in Table 1 suggested a ±2.5 ppm window centred on the theoretical exact masses of the metabolites to deliver extracted chromatograms that can be readily used for quantitation without significant isobaric interferences. Indeed, increasing the mass uncertainty windows did not increase the area-under-the-curve (AUC) of the investigated, well-resolved chromatography peaks, showing that the chosen 2.5 ppm windows captured all ions across the peak for those ions. For some isobaric ions, however, increasing the uncertainty allowance yielded the incorporation of other masses, as exemplified in Fig. 1. The figure shows that after increasing the ppm window to values >2.5 ppm, it was no longer possible to distinguish two important M + 22 species, generated by either sodiation or the addition of two carbons and a further unsaturation site. In our experiments, 2.5 ppm was considered the largest mass uncertainty window for quantitative ion extractions from plasma samples, as confirmed by a suggestion in the literature.^5^

Importantly, because the theoretical masses can be used for ion extraction, it becomes possible to query the data with a list of theoretical candidate metabolites, without the need for any prior experimental screening, results or evidence. Absolute quantitation will of course be obscured by the lack of standards and commonly observed problems such as ion suppression and other matrix effects. The real advantage of this fast scanning procedure, however, is the post-acquisition availability of accurate mass information for any ion in the full scan spectrum, with a degree of specificity equal to most MS/MS assays.^20^ Furthermore, although MS/MS is very frequently able to distinguish between isomers, it often has difficulty in separating compounds with isobaric masses or near isobaric masses, which can be separated by high-resolution, extracted ion chromatograms. For example, based on the theoretical *m/z* ratios of 50 phosphocholine lipids in our samples, we were able to extract 50 species based on their ion chromatograms (Table 2). The analytical quality of these results was assessed by determining the variance between the repeated analyses. Each sample was analysed four times (2 × injecting 10 µL and 2 × injecting 5 µL) and the coefficient of variance (CV) was determined calculated correcting for the injection volume. Measurements resulting in a CV of over 20% were rejected. On this basis the first four runs of the batch were rejected, as well as some of the larger phospholipids in some samples and the low-abundance sphingomyelins in most of the runs using 5 µL injections.

**Table 2:** The post-run targeted analysis of 50 phoshopholipids using their theoretical, exact masses. In three runs (#25, #150, #240) across the batch of 240 runs, the measured *m/z* ratios are reported as well as their deviation from the calculated theoretical *m/z*. In addition, the average measured *m/z* is given for 10 runs (#25, #50, #75, #100, #125, #150, #175, #200, #225 and #240)

		Run 25			Run 150			Run 240			
Lipid name	Calc. (*m/z*)	Rt (min)	*m/z*	Error (ppm)	Rt (min)	*m/z*	Error (ppm)	Rt	*m/z*	Error (ppm)	Average *m/z* (±stdev, n = 10)
**GPCho(14:0/0:0)**	468.3085	6.62	468.3087	0.4	6.61	468.3082	0.6	6.6	468.3084	0.2	468.3084 ± 0.0003
**GPEtn(18:1/0:0)**	480.3085	6.92	480.3088	0.6	6.9	480.3084	0.2	6.88	480.3091	1.2	480.3086 ± 0.0004
**GPCho(O-16:1)**	480.3449	7.01	480.3453	0.8	7	480.3447	0.4	6.98	480.3449	0.0	480.3449 ± 0.0005
**GPEtn(18:0/0:0)**	482.3241	6.75	482.3234	1.5	6.73	482.3235	1.2	6.72	482.3247	1.2	482.3239 ± 0.0004
**GPCho(O-16:0)**	482.3605	7.08	482.3602	0.6	7.06	482.36	1.0	7.04	482.36	1.0	482.3602 ± 0.0004
**GPCho(16:1/0:0)**	494.3241	6.67	494.3239	0.4	6.66	494.3241	0.0	6.65	494.3241	0.0	494.3242 ± 0.0002
**GPCho(16:0/0:0)**	496.3398	6.89	496.3398	0.0	6.87	496.3398	0.0	6.86	496.3398	0.0	496.3398 ± 0.0003
**GPEtn(20:4/0:0)**	502.2928	6.69	502.2932	0.8	6.68	502.293	0.4	6.67	502.293	0.4	502.2929 ± 0.0004
**GPCho(O-18:1)**	508.3762	7.10	508.3763	0.2	7.1	508.3755	1.4	7.09	508.3765	0.6	508.3760 ± 0.0005
**GPCho(18:3/0:0)**	518.3241	6.59	518.3243	0.4	6.57	518.3241	0.0	6.57	518.3239	0.4	518.3240 ± 0.0003
**GPCho(18:2/0:0)**	520.3398	6.72	520.3392	1.2	6.71	520.3396	0.4	6.7	520.3393	1.0	520.3397 ± 0.0004
**GPCho(18:1/0:0)**	522.3554	6.93	522.3554	0.0	6.91	522.3555	0.2	6.9	522.3557	0.6	522.3554 ± 0.0004
**GPCho(18:0/0:0)**	524.3711	7.22	524.3708	0.6	7.19	524.3715	0.8	7.18	524.3701	1.9	524.3709 ± 0.0005
**GPEtn(22:6/0:0)**	526.2938	6.64	526.2928	1.9	6.62	526.2929	1.7	6.61	526.2928	1.9	526.2930 ± 0.0003
**GPCho(20:5/0:0)**	542.3241	6.55	542.3240	0.2	6.54	542.3238	0.6	6.53	542.3236	0.9	542.3237 ± 0.0003
**GPCho(20:4/0:0)**	544.3398	6.69	544.3398	0.0	6.68	544.3399	0.2	6.67	544.3405	1.3	544.3397 ± 0.0005
**GPCho(20:3/0:0)**	546.3554	6.80	546.3552	0.4	6.79	546.3551	0.5	6.78	546.3545	1.6	546.3552 ± 0.0003
**GPCho(22:6/0:0)**	568.3398	6.65	568.3398	0.0	6.64	568.3396	0.4	6.63	568.3398	0.0	568.3399 ± 0.0003
**SM(d18:1/14:0)**	675.5436	6.9	675.5426	1.5	6.9	675.5432	0.6	6.83	675.5428	1.2	675.5433 ± 0.0007
**SM(d18:1/15:0)**	689.5592	6.69	689.5597	0.7	6.69	689.5599	1.0		nd		689.5594 ± 0.0008
**SM(d18:1/16:1)**	701.5592	6.97	701.5588	0.6	6.97	701.5587	0.7	6.69	701.5586	0.9	701.5588 ± 0.0004
**GPCho(O-34:3)**	742.5745	7.38	742.5746	0.1	7.37	742.5755	1.3	7.32	742.5739	0.8	742.5753 ± 0.0008
**GPCho(O-34:2)**	744.5902		nd		7.43	744.59	0.3	7.34	744.5899	0.4	744.5895 ± 0.0006
**GPCho(34:4)**	754.5381	6.97	754.5369	1.6		nd		6.81	754.5389	1.1	754.5379 ± 0.0010
**GPCho(34:3)**	756.5538	7.40	756.5548	1.3	7.39	756.5541	0.4	7.34	756.5533	0.7	756.5538 ± 0.0008
**GPCho(34:2)**	758.5694	6.89	758.5690	0.5	6.83	758.569	0.5	6.68	758.5701	0.9	758.5695 ± 0.0006
**GPCho(34:1)**	760.5851	7.23	760.5854	0.4	7.37	760.5854	0.4	7.37	760.5852	0.1	760.5855 ± 0.0004
**GPCho(O-36:6)**	764.5589	7.09	764.5590	0.1	7.05	764.5579	1.3		nd		764.5588 ± 0.0005
**GPCho(O-36:5)**	766.5745	7.41	766.5736	1.2	7.39	766.5743	0.3	7.33	766.5738	0.9	766.5740 ± 0.0008
**GPCho(O-36:3)**	770.6058	7.32	770.6055	0.4	7.3	770.6067	1.2	7.35	770.6067	1.2	770.6061 ± 0.0009
**GPCho(36:5)**	780.5538	7.38	780.5532	0.8	7.36	780.5537	0.1	7.32	780.5532	0.8	780.5537 ± 0.0010
**GPCho(36:4)**	782.5694	7.43	782.5703	1.2	7.39	782.5698	0.5	7.34	782.5692	0.3	782.5696 ± 0.0009
**GPCho(36:3)**	784.5851	7.37	784.5856	0.6	7.39	784.5851	0.0	7.33	784.5851	0.0	784.5845 ± 0.0009
**GPCho(36:2)**	786.6007	7.04	786.6006	0.1	7.04	786.6002	0.6	6.99	786.6005	0.3	786.6004 ± 0.0006
**GOCho(O-38:7)**	790.5745		nd		7.4	790.5748	0.4	7.32	790.5737	1.0	790.5743 ± 0.0008
**GPCho(O-38:6)**	792.5902	7.42	792.5895	0.9	7.39	792.5901	0.1	7.33	792.5902	0.0	792.5897 ± 0.0009
**GPCho(O-38:5)**	794.6058	7.40	794.6063	0.6	7.38	794.6064	0.8	7.32	794.605	1.0	794.6056 ± 0.0008
**GPCho(O-38:4)**	796.6215		nd		7.37	796.6219	0.5	7.36	796.6206	1.1	796.6218 ± 0.0009
**GPCho(38:7)**	804.5538	7.37	804.5524	1.7	7.19	804.5528	1.2		nd		804.5524 ± 0.0003
**GPCho(38:6)**	806.5694	7.39	806.5692	0.2	7.35	806.5694	0.0	7.35	806.5682	1.5	806.5690 ± 0.0009
**GPCho(38:5)**	808.5851	7.41	808.5857	0.7	7.39	808.585	0.1	7.33	808.5835	2.0	808.5846 ± 0.0008
**GPCho(38:4)**	810.6007	7.4	810.6013	0.7	7.42	810.6004	0.4	7.34	810.6003	0.5	810.6007 ± 0.0007
**GPCho(38:3)**	812.6164	7.4	812.6167	0.4	7.39	812.6174	1.2	nd	812.6174	1.2	812.6164 ± 0.0009
**GPCho(O-40:6)**	820.6215		nd		7.37	820.6201	1.7	7.32	820.6212	0.4	820.6214 ± 0.0012
**GPCho(40:7)**	832.5851	7.40	832.5851	0.0	7.37	832.5854	0.4	7.52	832.5836	1.8	832.5844 ± 0.0010
**GPCho(40:6)**	834.6007	7.14	834.6013	0.7	7.08	834.6006	0.1		nd		834.6006 ± 0.0010
**GPCho(40:5)**	836.6164	7.4	836.6169	0.6		nd		7.35	836.6147	2.0	836.6170 ± 0.0011

Lipid nomenclature was used according to Lipid Maps.^21^

nd: not detected; Rt: retention time.

**Figure 2:**
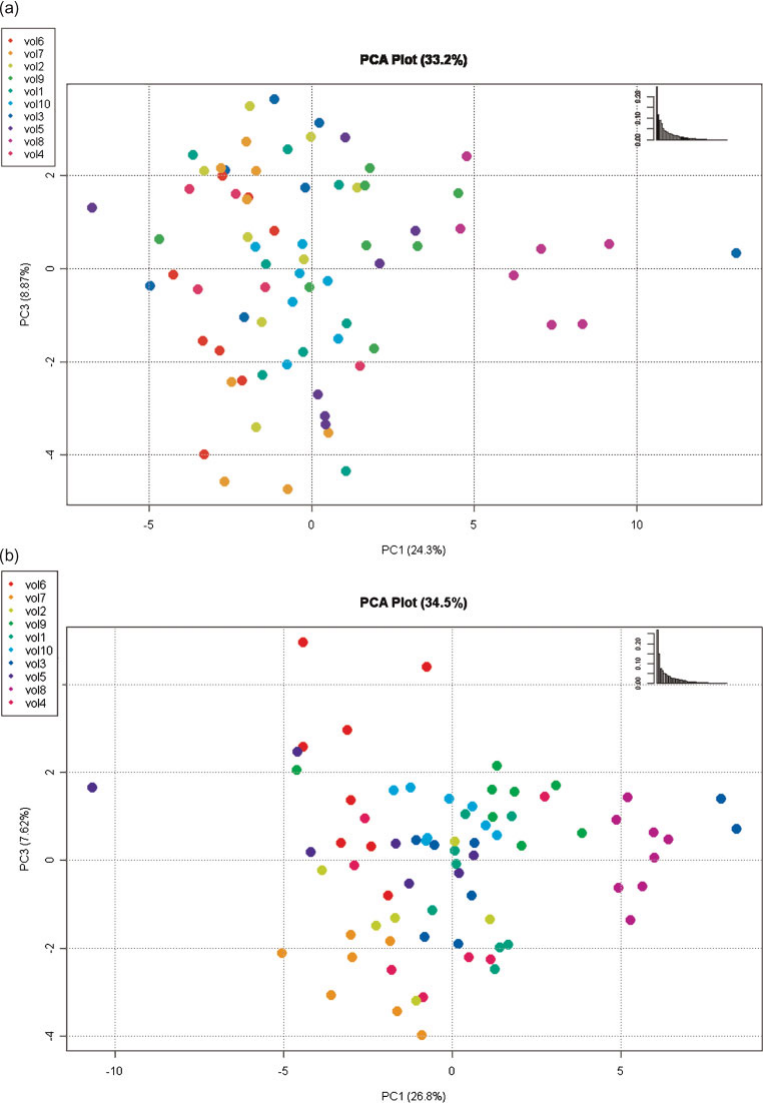
PCA based on (a) 5 µL and (b) 10 µL injection using post-run targeted analysis of 50 phospholipids with six samples per volunteers; showing PC1 and PC3 with the eigenvalues in brackets. (The labels refer to different volunteers from whom the plasma samples originated.) This figure is available in colour online at http://www.interscience.wiley.com/journal/rcm

We also investigated whether the different levels of these compounds can be used to differentiate between the different human volunteers. Integrated peak values were used in these experiments and exported into DANTE.^17^ (Analytical runs using 5 µL injections and 10 µL injections were analysed separately.) Missing data values were imputed using the k Nearest Neighbour method. The resulting data were analysed by PCA (Fig. 2). Both PCA plots show a similar distribution (using PC1 and PC3). The main difference between injection volumes is that the samples from volunteers 6 and 7 were clearly separated by PC3 in the 10 µL injection series, but this separation was lost with the 5 µL injections. The reason for this phenomenon is that the predominantly contributing phospholipids (SM(d18:1/16:1) and SM(d18:1/15:0)) were only minor constituents, mostly not quantifiable in the 5 µL injection experiments. The loadings contributing to both PCAs are very similar for four out of the top five, the same for the 5 µL and 10 µL injections.

### Unbiased metabolomics

**Figure 3:**
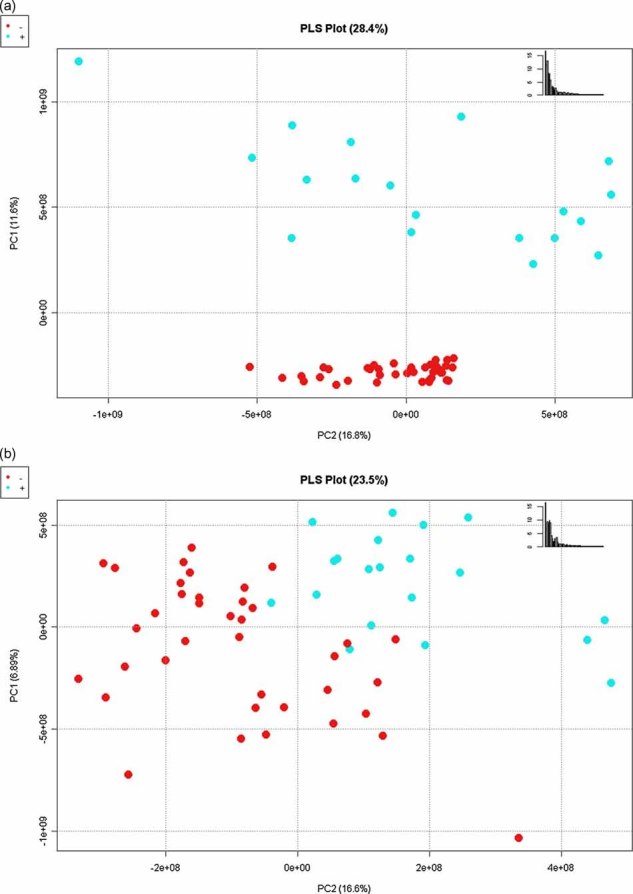
(a) PLS-DA analysis of samples fortified with the ceramide N-octanoylsphingosine, based on unsupervised data extraction using MZMine; showing PC1 and PC2 with the eigenvalues in brackets. (+ samples fortified with N-octanoylsphingosine; – samples not fortified with N-octanoylsphingosine.) (b) PLS-DA analysis of samples fortified with the ceramide glucose, based on unsupervised data extraction using MZMine; showing PC1 and PC2 with the eigenvalues in brackets. (+: samples fortified with glucose; –: samples not fortified with glucose.) This figure is available in colour online at http://www.interscience.wiley.com/journal/rcm

The experimental data were also ideally suited to analysis by standard unsupervised metabolomics approaches. To demonstrate this, the 240 data files were converted into the NetCDF format and imported into the open-source software package MZmine.^18^ This program was used for peak-picking and peak alignment. The obtained data were exported as spreadsheets. By using principle component and partial least-squares (PLS) analyses (see Fig. 3), we were able to readily identify the different persons from which the blood samples originated as well as the different fortifications employed to some of the samples (fortification refers to the addition of several compounds from different chemical classes to aliquots of the plasma samples). The fortification was conducted to retain the normal biological variation of these samples but have surrogate markers allowing classification using unbiased profiling. Table 3 illustrates the top seven loadings from the PLS analysis (see Fig. 3), showing clear difference between the ceramide-fortified samples and plasma samples without ceramide addition. The *m/z* ratios of these ions yielded molecular formulae for protonated molecules after water loss, protonated molecules, as well as sodiated species, etc. The same approach was used for samples fortified with glucose. The separation using PLS between the two groups was less clear than for ceramide fortification, as glucose was already present in the plasma samples, leading only to elevated levels for the fortified sample group.

**Table 3:** Loadings from the first component from PCA (see Fig. 3(a))

Measured (*m/z*)	Rt (s)	Loading	Mol formulae	Theoretical (*m/z*)	Identity
408.3834	395.4	0	C_26_H_50_O_2_N^+^	408.38361	(Cer-H_2_O) H^+^
448.3760	396.6	0.0002	C_26_H_51_O_3_NNa^+^	448.37667	(Cer) Na^+^
426.3940	396.8	0.0006	C_26_H_52_O_3_N^+^	426.39445	(Cer) H^+^
873.7631	396.6	0.0008	C_52_H_102_O_6_N_2_Na^+^	873.76300	2(Cer) Na^+^
409.3868	396.9	0.0010	C_25_^13^CH_50_O_2_N^+^	409.38696	(Cer-H_2_O) H^+^-isotope
874.7664	396.5	0.0012	C 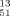 CH_102_O_6_N_2_Na^+^	874.76636	2(Cer)Na^+^-isotope
449.3794	396.2	0.0014	C 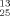 CH_51_O_2_NNa^+^	449.37947	(Cer) Na^+^-isotope

Cer: N-octanoylsphingosine.

The two approaches described in this study exhibit both advantages and disadvantages. Using selective *m/z* ratios is more powerful for distinguishing quantitative differences of known compounds, while unsupervised profiling is able to show qualitative differences, for known and unknown components. The use of high-resolution mass spectrometry for metabolomics offers the possibility for both approaches without compromises; complementary information is obtained thus circumventing any errors in peak-picking and alignment, which are always present. The use of a target list can also exclude ions for which poor quantitative measurements are expected due to sample preparation or chromatography.

## CONCLUSIONS

Based on the initial set of experiments described in this study, the new benchtop orbitrap instrument offers great potential for the development of new powerful approaches in metabolomics and lipidomics. We have shown that acquired high-resolution data can be used in different approaches, targeted and untargeted, at the same time, without compromises in analytical quality, which so far was not possible in a single assay. We envision the incorporation of specific mass defects or software tools using databases for post-run analysis of the data for specific metabolites in the future. Furthermore, the use of simultaneous MS/MS data will greatly enhance analytical specificity, however, at the price of reduced scan speeds. Future developments of the orbitrap mass analyser have to address the trade-off between resolving power and scan speed, as increasing the resolving power significantly above >50 000 comes at the price of significantly slower scan speeds, not compatible with fast chromatography anymore.

## References

[1] Loftus N, Miseki K, Iida J, Gika HG, Theodoridis G, Wilson ID (2008). Rapid Commun. Mass Spectrom..

[2] Plumb RS, Johnson KA, Rainville P, Smith BW, Wilson ID, Castro-Perez JM, Nicholson JK (2006). Rapid Commun. Mass Spectrom..

[3] Smith CA, Want EJ, O'Maille G, Abagyan R, Siuzdak G (2006). Anal Chem..

[4] Nielsen J, Jewett MC (2007). Topics Curr. Genet..

[5] Schwudke D, Hannich JT, Surendranath V, Grimard V, Moehring T, Burton L, Kurzchalia T, Shevchenko A (2007). Anal. Chem..

[6] Want EJ, O'Maille G, Smith CA, Brandon TR, Uritboonthai W, Qin C, Trauger SA, Siuzdak G (2006). Anal. Chem..

[7] Nielsen J, Oliver S (2005). Trends Biotechnol..

[8] van der Werf MJ, Overkamp KM, Muilwijk B, Coulier L, Hankemeier T (2007). Anal. Biochem..

[9] Colombo M, Sirtori FR, Rizzo V (2004). Rapid Commun. Mass Spectrom..

[10] Eckers C, Wolff JC, Haskins NJ, Sage AB, Giles K, Bateman R (2000). Anal. Chem..

[11] Charles L (2003). Rapid Commun. Mass Spectrom..

[12] Pelander A, Ojanpera I, Laks S, Rasanen I, Vuori E (2003). Anal. Chem..

[13] Marshall AG, Rodgers RP (2004). Acc. Chem. Res..

[14] Stenson AC, Marshall AG, Cooper WT (2003). Anal. Chem..

[15] Breitling R, Pitt AR, Barrett MP (2006). Trends Biotechnol..

[16] Sleno L, Volmer DA, Marshall AG (2005). J. Am. Soc. Mass Spectrom..

[17] Polpitiya AD, Qian WJ, Jaitly N, Petyuk VA, Adkins JN, Camp DG, Anderson GA, Smith RD (2008). Bioinformatics.

[18] Katajamaa M, Miettinen J, Oresic M (2006). Bioinformatics.

[19] Hu C, van Dommelen J, van der Heijden R, Spijksma G, Reijmers TH, Wang M, Slee E, Lu X, Xu G, van der Greef J, Hankemeier T (2008). J. Proteome Res..

[20] Merrill AH, Sullards MC, Allegood JC, Kelly S, Wang E (2005). Methods.

[21] http://www.lipidmaps.org.

